# Joint Optimization of Interference Coordination Parameters and Base-Station Density for Energy-Efficient Heterogeneous Networks

**DOI:** 10.3390/s19092154

**Published:** 2019-05-09

**Authors:** Yanzan Sun, Han Xu, Shunqing Zhang, Yating Wu, Tao Wang, Yong Fang, Shugong Xu

**Affiliations:** Shanghai Institute for Advanced Communication and Data Science, Key laboratory of Specialty Fiber Optics and Optical Access Networks, Joint International Research Laboratory of Specialty Fiber Optics and Advanced Communication, Shanghai University, Shanghai 200072, China; yanzansun@shu.edu.cn (Y.S.); xuhan_shu@shu.edu.cn (H.X.); shunqing@shu.edu.cn (S.Z.); ytwu@shu.edu.cn (Y.W.); yfang@shu.edu.cn (Y.F.); shugong@shu.edu.cn (S.X.)

**Keywords:** HetNets, interference coordination, energy efficiency, stochastic geometry

## Abstract

Heterogeneous networks (HetNets), consisting of macro-cells and overlaying pico-cells, have been recognized as a promising paradigm to support the exponential growth of data traffic demands and high network energy efficiency (EE). However, for two-tier heterogeneous architecture deployment of HetNets, the inter-tier interference will be challenging. Time domain further-enhanced inter-cell interference coordination (FeICIC) proposed in 3GPP Release-11 becomes necessary to mitigate the inter-tier interference by applying low power almost blank subframe (ABS) scheme. Therefore, for HetNets deployment in reality, the pico-cell range expansion (CRE) bias, the power of ABS and the density of pico base stations (PBSs) are three important factors for the network EE improvement. Aiming to improve the network EE, the above three factors are jointly considered in this paper. In particular, we first derive the closed-form expression of the network EE as a function of pico CRE bias, power reduction factor of low power ABS and PBS density based on stochastic geometry model. Then, the approximate relationship between pico CRE bias and power reduction factor is deduced, followed by a linear search algorithm to get the near-optimal pico CRE bias and power reduction factor together at a given PBS density. Next, a linear search algorithm is further proposed to optimize PBS density based on fixed pico CRE bias and power reduction factor. Due to the fact that the above pico CRE bias and power reduction factor optimization and PBS density optimization are optimized separately, a heuristic algorithm is further proposed to optimize pico CRE bias, power reduction factor and PBS density jointly to achieve global network EE maximization. Numerical simulation results show that our proposed heuristic algorithm can significantly enhance the network EE while incurring low computational complexity.

## 1. Introduction

The exponential growth of data traffic demand, huge energy consumption and large amounts of global carbon dioxide emissions severely restrict the sustainable development of wireless cellular networks. According to the statistics, the data traffic volume demand in the fifth-generation (5G) wireless communication network will increase of 1000× by 2020. Moreover, the limited spectrum resources also constrain the network capacity improvement [1]. Therefore, the network energy efficiency (EE) which considers both spectral efficiency (SE) and energy consumption has been valued not only as an important network performance indicator for modern wireless networks, but also for the operational expenditure reduction and sustainable development [2].

Heterogeneous networks (HetNets) consisting of macro base stations (MBSs) and low-power pico base stations (PBSs) can improve SE by reusing the spectrum geographically [3] and enhance the wireless link quality by shortening the distance between the transmitter and the receiver [4]. Therefore, HetNets are deemed as a promising technique to support the deluge of data traffic with high network EE. Nonetheless, due to the complex two-tier heterogeneous architecture deployment of HetNets, the challenging inter-tier interference and PBS deployment density will deteriorate the network EE if they are not treated carefully, which are the concerns of this paper for aiming to improve the network EE.

### 1.1. Motivation

**Figure 1:**
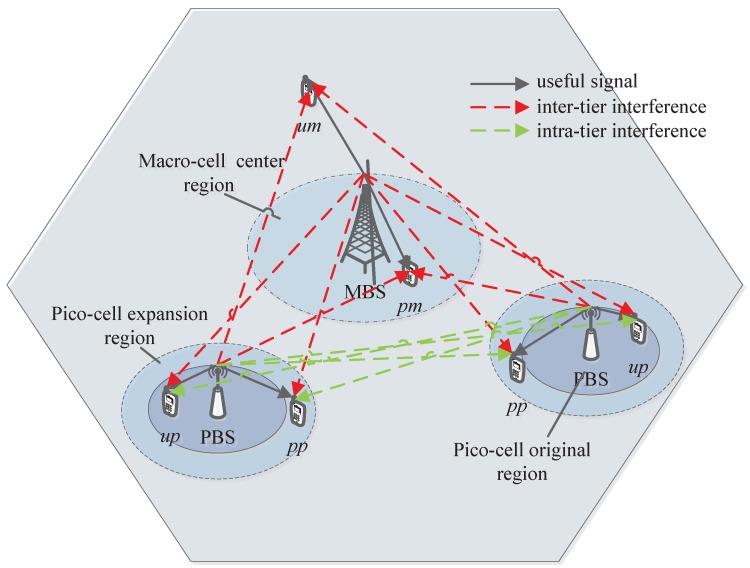
The network scenario.

In HetNets consisting of MBSs and PBSs, as shown in Figure 1, the great difference of transmitter power leads to load imbalance between macro tier and pico tier. To address this issue, PBSs adopt cell range expansion (CRE) technology to enlarge the PBS coverage area by adding a positive bias on the reference signal received power (RSRP) of PBSs without increasing transmission power [5,6]. Unfortunately, CRE user equipments (UEs) located in the pico CRE region will suffer serious downlink interference from dominating MBSs, even causing the outage of control signals. As a result, it is important to mitigate the downlink interference to improve the wireless link quality between transmitter and receiver. Then the network EE can be improved.

To mitigate the downlink inter-tier interference for HetNets, further enhanced inter-cell interference coordination (FeICIC) scheme has been specified in 3GPP Release 11 [7]. In FeICIC scheme, MBSs can transmit data to macro center UEs with low transmission power over certain subframes, termed as low power almost blank subframes (ABS), over which PBSs can schedule CRE UEs with reduced interference [8]. On the basis of FeICIC technique, for user association, the pico CRE bias will directly affect the value of user received RSRP from PBSs, which will eventually affect the number of CRE UEs associated to PBSs. As CRE UEs are scheduled in the subframes corresponding to the ABS of MBSs, the transmission power of MBSs in ABS, i.e., ABS power, will decide the interference degree suffered by CRE UEs. Therefore, pico CRE bias and ABS power are two important factors for the wireless link quality of UEs, especially for CRE UEs, which eventually have significant effect on the network EE performance.

To meet the super-large capacity demand in 5G wireless communication networks, more and more base stations (BSs), especially small base stations (SBSs), are deployed in HetNets [9,10]. On the one hand, irregular deployment of massive BSs causes additional and intractable inter-tier interference. On the other hand, although high-density PBSs are deployed to satisfy the peak traffic volume, highly dynamic wireless traffic may deteriorate EE if the capacity gains of numerous PBSs are utilized insufficiently. In short, the BSs deployment strategy based on network load is also one of the key issues to realize 5G green cellular network [11].

In addition, the network EE of HetNets is also affected by some other aspects of factors. For instance, it is proved that the reasonable adjustments of BS transmit power, inter-site distances and the number of MBSs or SBSs contribute to the improvement of EE of HetNets [12]. In [13], assuming that SBSs have performed traffic offloading from MBS, the authors investigated the MBS and SBS power allocation scheme to improve network EE. In [14], the authors investigated the user scheduling and resource allocation method to optimize the network EE for HetNets.

To sum up, for complex HetNets, network EE is affected by many different aspects of factors, e.g., MBS transmit power in low power ABS, MBS transmit power in non-ABS, MBS density, ABS ratio, pico CRE bias, PBS density, PBS transmit power in low power ABS, PBS transmit power in non-ABS, etc. Thus, it will be a very challenging work to analyze the effects of all of these factors together on network EE. In this paper, for HetNets deployment in reality with inter-tier interference coordination by adopting FeICIC scheme, the pico CRE bias, the power reduction factor of low power ABS, and the density of PBSs are three more related factors for the network EE improvement compared with others. Therefore, the above three factors are focused and jointly optimized for the network EE maximization in this paper.

### 1.2. Contributions

In this paper, we investigate the joint optimization of FeICIC parameters and PBS density in HetNets for network EE improvement. Initially, the system model for two-tier HetNets by using stochastic geometry is described. Then, an analytical expression of the network EE as a function of pico CRE bias, power reduction factor and PBS density is derived. At last, heuristic algorithms are proposed to obtain the optimal values of pico CRE bias, power reduction factor and PBS density to maximize the network EE. The main contributions of this paper are summarized as follows: (1) the closed-form expression of network EE as a function of pico CRE bias, power reduction factor and PBS density is deduced by stochastic geometry theory. (2) The equivalent relationship between pico CRE bias and the power reduction factor is obtained by an approximation calculation. Based on the equivalent relationship, an efficient optimization algorithm is designed to get the near-optimal values of pico CRE bias and power reduction factor at a given PBS density. (3) To achieve the global optimization of network EE, a low computational complexity heuristic algorithm is proposed to jointly optimize pico CRE bias, power reduction factor, and PBS density.

### 1.3. Organization

The rest of this paper is organized as follows. The system model and user association strategy are described in Section 3. In Section 4, we deduce the closed-form expression of network EE. A low complexity heuristic algorithm is proposed to optimize pico CRE bias, power reduction factor, and PBS density jointly for the network EE maximization in Section 5. Numerical results and discussions are presented in Section 6. Concluding remarks are given in Section 7.

## 2. Related Works

Early literatures mainly focused on pico CRE bias, ABS ratio, and ABS power optimization for the network capacity maximization [15,16,17,18] and recent works began to investigate the network EE improvement from different perspectives including resource management [19,20,21,22,23], FeICIC parameters optimization [24,25,26,27,28,29,30,31] and BS deployment strategy [32,33,34,35,36,37,38,39]. As for resource management, centralized resource allocation algorithms based on convex-optimization [19,20], graph-theory [21] or even game-theory [22,23] were proposed to achieve the maximum EE gain.

As for FeICIC parameter optimization, the ABS ratio dynamical optimization algorithm based on network load was proposed to enhance the network EE in [24]. For improving the network capacity and EE, pico CRE bias optimization problem was further developed combined with power control in [29]. Using the stochastic geometric approach, the expressions for SE and cell-edge throughputs have been derived as a function of the power reduction factor of low power ABS in [30]. To move one step further, pico CRE bias, ABS ratio and ABS power are jointly optimized by a robust EE optimization framework in [25]. In [31], it was proved that FeICIC can achieve a better gain in view of network EE, cell-edge throughput and user fairness compared with eICIC. In addition, the distributed algorithms based on the exact potential game framework for both eICIC and FeICIC optimizations were proposed to offer better network performance. The authors of [26] deducted and analyzed the EE coverage performance of FeICIC with extra pico CRE bias. In [28], FeICIC technique was applied to mitigate the inter-tier interference in HetNets deployed with unmanned aerial vehicles (UAVs). In such a scenario, the locations of UAVs, pico CRE bias and inter-cell interference coordination parameters were jointly optimized by using a genetic algorithm. In [27], the EE of HetNets with joint FeICIC and adaptive spectrum allocation was analyzed by the stochastic geometric approach. The research on FeICIC parameters optimization on the basis of stochastic geometry is relatively few in the existing literatures.

As for BS deployment strategy, general linear power consumption models were developed by means of linear regression in [38]. Meanwhile, the effects of MBS transmition power and on/off switching on instantaneous MBS power consumption were analyzed. A threshold of SBS density in ultra-dense HetNets was investigated in consideration of the backhaul network capacity and network EE in [32]. In [33], the authors came up with an approximation algorithm to solve the intractable user association problem by controlling the PBS density dynamically. The relationship between PBS density and network EE was analyzed under different UE density in [34]. It was proved that both PBS density and MBS density have a notable impact on the network EE in [35]. In [36,37], PBS density and MBS density were jointly optimized based on traffic-aware sleeping strategies and stochastic geometry to enhance the network EE, respectively. In [39], taking the traffic pattern variations into account, the BSs can not only adaptively switch on/off states but also can dynamically scale its transmit power according to network capacity demands. In this way, network energy consumption is reduced.

Despite the aforementioned research works, few works in the literatures focused on FeICIC parameters and PBS density joint optimization for network EE improvement in HetNets, which will be investigated and developed in this paper.

## 3. System Model

The traditional network models with a hexagonal grid cannot accurately match the actual network deployment. Under such deterministic grid models, Monte Carlo simulations consume huge amounts of time and resources to obtain the statistical results. Recently, a stochastic geometry model was proven to be a tractable analytical model for homogeneous networks and HetNets, where the location distribution of BSs is modeled as a spatial Poisson point process (PPP) [40]. Using PPP, the network performance, like signal to interference plus noise ratio (SINR) coverage [41], rate coverage [8], average rate [42], can be analyzed conveniently by theoretical derivation. Thus, we adopt a stochastic geometry model to model a two-tier HetNets consisting of MBSs and PBSs in this paper.

Let $k\in \left\{m,p\right\}$ denote the subscripts of a tier, where k=m represents macro tier and k=p denotes pico tier. MBSs and PBSs are modeled as two identically independent distributions (iid.) PPPs $\Phi_{m}$ and $\Phi_{p}$ with density $\lambda_{m}$ and $\lambda_{p}$ in the Euclidean plane, respectively. The UEs are also distributed according to a different iid PPP $\Phi_{u}$ with density $\lambda_{u}$. The total spectrum bandwidth is defined as *W*. To mitigate the downlink interference over the control channel from MBSs to CRE UEs, MBSs adopt the FeICIC scheme, where all the subframes are divided into protected subframes (PSF), i.e., low power ABS, and unprotected subframes (USF), i.e., non-ABS. The MBSs transmit data at reduced power $\rho P_{m}$ on PSF, where 0≤ρ<1 is the power reduction factor. In fact MBS transmit power in USF and PBS transmit power will also have effects on network EE. However, to focus on the effects of PBS density, pico CRE bias and power reduction factor on network EE and also for analysis simplification, we assume that MBSs transmit power in USF and PBSs transmit power are set to be the maximum fixed power $P_{m}$ and $P_{p}$, respectively. Let θ to be PSF ratio, which is defined as the proportion between the number of PSF subframes and that of all subframes. Each user is associated with the strongest BS according to the biased received reference signal received power (RSRP) at the user. In this paper, the association bias for MBS is assumed to be unity ($B_{m}=1=0\;\mathrm{dB}$) and that of PBS is pico CRE bias depicted as $B_{p}$, where $B_{p}\geq 0\;\mathrm{dB}$.

**Figure 2:**
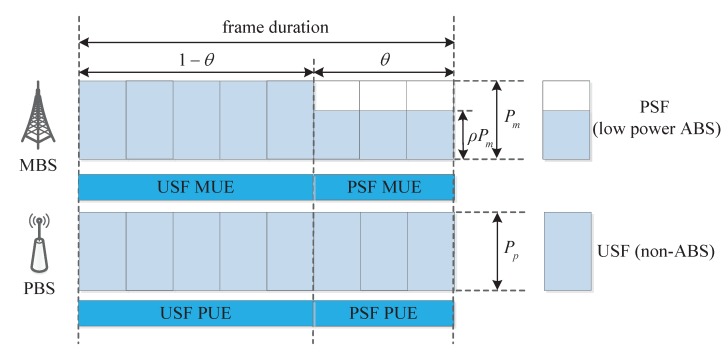
The user equipments (UEs) scheduling strategy for macro base station (MBS) and pico base stations (PBS) with the further-enhanced inter-cell interference coordination (FeICIC) scheme.

Based on the user association strategy, all UEs can be divided into four different types: the type of PSF macro-cell UEs (MUEs) contains the users within the macro-cell center region, the type of USF MUEs includes the users outside the macro-cell center region, the type of PSF pico-cell UEs (PUEs) correspond the users located in the pico CRE region and the type of USF PUEs comprises the users scattered in the original coverage of pico-cell. As shown in Figure 1, we adopt the index $l\in L=\left\{pm,um,pp,up\right\}$ to denote the indication of the above four types of users, respectively, where pm represents PSF MUEs, um denotes USF MUEs, pp signifies PSF PUEs, and up indicates USF PUEs. In HetNets with FeICIC, the UEs scheduling strategy for MBS and PBS is shown in Figure 2. The USF MUEs and USF PUEs are scheduled by MBSs and PBSs on USF, respectively. Each MBS schedules PSF MUEs on PSF with reduced power. Then PBS can schedule PSF PUEs in the corresponding subframes with reduced interference.

According to the Slivyak theorem, there is no difference in properties observed either at a point of the PPP or at an arbitrary point [8]. Therefore, we can simply analyze a typical UE located at the origin. The received signal power of a typical user *l* from a BS of *k* tier at a distance of $r_{k}$ can be represented as $P_{k}hr_{k}^{-\alpha }$, where $P_{k}$ is the full transmission power of BS in *k* tier, *h* represents the channel fast fading gain, which is defined as Rayleigh distributed with average unit power, i.e., h∼exp(1). The term α denotes the large-scale path loss exponent, which is assumed to be the same in both macro tier and pico tier for convenient analysis. Hence, the SINR of a typical user *l* based on its user type can be depicted as:

(1) $$\gamma_{l}=\left\{\begin{array}{c} \frac{\rho P_{m}hr_{m}^{-\alpha }}{\rho I_{m}+I_{p}+\sigma^{2}},\mathrm{if}\;l=pm\\ \frac{P_{p}hr_{p}^{-\alpha }}{\rho I_{m}+I_{p}+\sigma^{2}},\mathrm{if}\;l=pp\\ \frac{P_{m}hr_{m}^{-\alpha }}{I_{m}+I_{p}+\sigma^{2}},\mathrm{if}\;l=um\\ \frac{P_{p}hr_{p}^{-\alpha }}{I_{m}+I_{p}+\sigma^{2}},\mathrm{if}\;l=up \end{array}\right.,$$

where $I_{m}$ and $I_{p}$ denote the full power aggregate interference from the macro tier and pico tier to UE *l*, respectively, $\sigma^{2}$ represents the thermal noise, ρ is the power reduction factor of MBS transmit power in PSF. $P_{m}$ and $P_{p}$ are the full transmission power of MBSs and PBSs, respectively. $r_{m}$ and $r_{p}$ are the nearest distance from MBSs and PBSs to a typical UE *l*, respectively.

The notations used in this paper are shown in Table 1.

### 3.1. User Type Probability

Normally, the user type of a typical UE *l* can be decided by the relationship between the biased RSRP from its nearest MBS and its nearest PBS as follow:

(2) $$l=\left\{\begin{array}{c} pm,\mathrm{if}\;\rho P_{m}hr_{m}^{-\alpha }>B_{p}P_{p}hr_{p}^{-\alpha }\\ pp,\mathrm{if}\;P_{p}hr_{p}^{-\alpha }<P_{m}hr_{m}^{-\alpha }<B_{p}P_{p}hr_{p}^{-\alpha }\\ um,\mathrm{if}\;\rho P_{m}hr_{m}^{-\alpha }<B_{p}P_{p}hr_{p}^{-\alpha }<P_{m}hr_{m}^{-\alpha }\\ up,\mathrm{if}\;P_{m}hr_{m}^{-\alpha }<P_{p}hr_{p}^{-\alpha } \end{array}\right.,$$

where $B_{p}$ is the pico CRE bias. In Equation (2), the conditions for determining user type can be further translated from biased RSRP based inequation into distance based inequation, which is shown as Equation (3).

(3) $$l=\left\{\begin{array}{c} pm,\mathrm{if}\;k_{c}r_{m}<r_{p}\\ pp,\mathrm{if}\;k_{p}r_{m}<r_{p}<k_{e}r_{m}\\ um,\mathrm{if}\;k_{e}r_{m}<r_{p}<k_{c}r_{m}\\ up,\mathrm{if}\;k_{p}r_{m}>r_{p} \end{array}\right.,$$

where $k_{c}={\left[B_{p}P_{p}/\left(P_{m}\rho \right)\right]}^{1/\alpha }$, $k_{e}={\left(B_{p}P_{p}/P_{m}\right)}^{1/\alpha }$ and $k_{p}={\left(P_{p}/P_{m}\right)}^{1/\alpha }$ are defined as macro-cell center region factor, pico CRE region factor and pico-cell original coverage region factor, respectively [8], determining the coverage bound of the macro-cell center region, the pico CRE coverage region and the pico-cell original coverage region, respectively.

In order to obtain the probabilities of four user types, the following lemma is proposed.

**Lemma** **1.** *Due to the locations of MBSs and PBSs follow two iid. PPPs, given two arbitrary coefficient values of $n_{a}$ and $n_{b}$, the probability of $n_{a}r_{m}<r_{p}<n_{b}r_{m}$ can be expressed as:*

(4) $$\begin{array}{c} \mathrm{Prob}\left(n_{a}r_{m}<r_{p}<n_{b}r_{m}\right)=\mathrm{Prob}\left(r_{p}>n_{a}r_{m}\right)-\mathrm{Prob}\left(r_{p}>n_{b}r_{m}\right)\\ \;\;\;\;\;\;\;\;\;\;\;\;\;\;\;\;\;\;\;\;\;\;\;\;\;\;\;\;\;\;\;\;\;\;\;\;\;\;\;\;=\frac{\lambda_{m}}{\lambda_{m}+n_{a}^{2}\lambda_{p}}-\frac{\lambda_{m}}{\lambda_{m}+n_{b}^{2}\lambda_{p}}, \end{array}$$

*where $\lambda_{m}$ and $\lambda_{p}$ are the MBS density and PBS density.*

**Proof.**  The proof of Lemma 1 is presented in Appendix A. □

Then, the probabilities of the PSF MUEs and the USF PUEs can be calculated similarly based on Lemma 1 and can be expressed by $\mathrm{Prob}\left(r_{p}>n_{a}r_{m}\right)=\frac{\lambda_{m}}{\lambda_{m}+n_{a}^{2}\lambda_{p}}$ and $\mathrm{Prob}\left(r_{p}<n_{b}r_{m}\right)=1-\mathrm{Prob}\left(r_{p}>n_{b}r_{m}\right)=1-\frac{\lambda_{m}}{\lambda_{m}+n_{b}^{2}\lambda_{p}}$, respectively. Combining Equation (4) with the user association strategy in Equation (3), the probability of this typical UE belonging to the user type *l* can be defined as $A_{l}=\mathrm{Prob}\left(l\in L\right)$, which is expressed as:

(5) $$A_{l}=\left\{\begin{array}{c} \frac{\lambda_{m}}{\lambda_{m}+k_{c}^{2}\lambda_{p}},\mathrm{if}\;l=pm\\ \frac{\lambda_{m}\lambda_{p}\left(k_{e}^{2}-k_{p}^{2}\right)}{\left(\lambda_{m}+k_{p}^{2}\lambda_{p}\right)\left(\lambda_{m}+k_{e}^{2}\lambda_{p}\right)},\mathrm{if}\;l=pp\\ \frac{\lambda_{m}\lambda_{p}\left(k_{c}^{2}-k_{e}^{2}\right)}{\left(\lambda_{m}+k_{e}^{2}\lambda_{p}\right)\left(\lambda_{m}+k_{c}^{2}\lambda_{p}\right)},\mathrm{if}\;l=um\\ \frac{k_{p}^{2}\lambda_{p}}{\lambda_{m}+k_{p}^{2}\lambda_{p}},\mathrm{if}\;l=up \end{array}\right..$$

**Figure 3:**
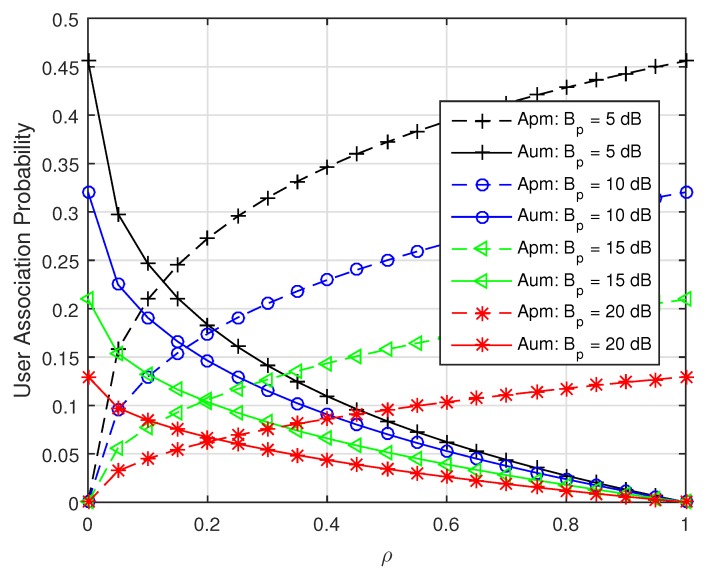
The user association probability of unprotected subframes (USF) micro-cell user equipments (MUEs) and protected subframes (PSF) MUEs versus ρ with fixed $\lambda_{p}=3\lambda_{m}$ and fixed $\lambda_{u}=0.0018$.

In particular, if ρ=0, then $k_{c}=\infty$ and $A_{pm}=0$, which means that the PSFs are zero power ABS. The association probabilities of USF MUEs and PSF MUEs versus ρ with different $B_{p}$ are simulated according to Equation (5) in Figure 3. As shown in Figure 3, with the increase of power reduction factor ρ, the transmission power of MBSs over PSF will increase, resulting in the association probability of USF MUEs decreasing and that of PSF MUEs increasing. It can also be found that the sum of $A_{pm}$ and $A_{um}$ will decrease with the growth of $B_{p}$, because more UEs will be offloaded into pico-cells with larger $B_{p}$.

### 3.2. Distribution of Serving BS Distance

After a typical UE type is classified according to the user association strategy, the probability density function (PDF) of distance *r* between this typical UE and its serving BS can be obtained according to Lemma 2 as below.

**Lemma** **2.** *On the basis of user association probability deduced in Equation (5), the probability density function (PDF) $f_{l}\left(r\right)$ of the distance r between the typical UE l and its serving BS can be derived as:*

(6) $$f_{l}\left(r\right)=\left\{\begin{array}{c} \frac{2\pi r\lambda_{m}}{A_{pm}}\exp\left[-\pi r^{2}\left(\lambda_{m}+k_{c}^{2}\lambda_{p}\right)\right],if\;l=pm\\ \frac{2\pi r\lambda_{p}}{A_{pp}}\left\{\exp\left[-\pi r^{2}\left(\lambda_{m}/k_{e}^{2}+\lambda_{p}\right)\right]-\exp\left[-\pi r^{2}\left(\lambda_{m}/k_{p}^{2}+\lambda_{p}\right)\right]\right\},if\;l=pp\\ \frac{2\pi r\lambda_{m}}{A_{um}}\left\{\exp\left[-\pi r^{2}\left(\lambda_{m}+k_{e}^{2}\lambda_{p}\right)\right]-\exp\left[-\pi r^{2}\left(\lambda_{m}+k_{c}^{2}\lambda_{p}\right)\right]\right\},if\;l=um\\ \frac{2\pi r\lambda_{p}}{A_{up}}\exp\left[-\pi r^{2}\left(\lambda_{m}/k_{p}^{2}+\lambda_{p}\right)\right],if\;l=up \end{array}\right..$$

**Proof.**  The proof is shown in Appendix B. □

## 4. Derivation of Energy Efficiency Expression

This section introduces our main analysis model and derives the closed-form expressions of the average achievable downlink rate, the network power consumption and the network EE, respectively.

### 4.1. The Ratio of PSF

PSF ratio can be denoted to be the proportion between the association probability of PSF PUE and the sum of the association probability of PSF PUE and that of USF PUE, as shown in Equation (7).

(7) $$\theta =\frac{A_{pp}}{A_{pp}+A_{up}}=\frac{\lambda_{m}\left(k_{e}^{2}-k_{p}^{2}\right)}{k_{e}^{2}\left(\lambda_{m}+k_{p}^{2}\lambda_{p}\right)},$$

where the expressions of $A_{pp}$ and $A_{up}$ are obtained according to Equation (5).

**Figure 4:**
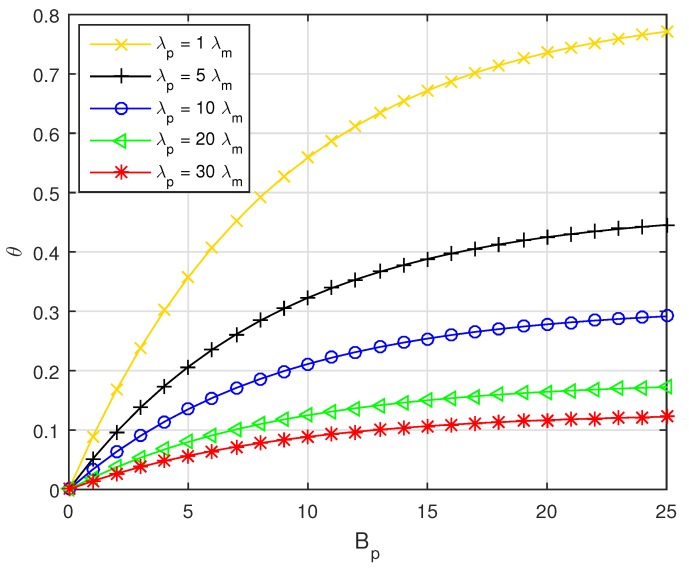
The PSF ratio versus $B_{p}$ with fixed $\lambda_{u}=0.0018$.

Referring to Equation (7), the PSF ratio versus $B_{p}$ with different PBS densities $\lambda_{p}$ is depicted in Figure 4. As shown in Figure 4, with $B_{p}$ increasing, the pico CRE area will be enlarged. As a result, the PSF ratio will rise. Moreover, with the PBS density $\lambda_{p}$ increasing, the distance between PBSs will be smaller, which will limit the further expansion of pico CRE area, so that the effect of $B_{p}$ on θ will be weakened with $\lambda_{p}$ increasing.

### 4.2. Average Achievable Downlink Rate

Assume that the network system adopts full buffer model and the frequency resource is allocated to all UEs in the coverage of a BS equally. Thus, the mean achievable downlink data rate of a typical UE *l* can be denoted as:

(8) $$R_{l}=\frac{W_{l}}{E\left[N_{l}\right]}E\left[\log_{2}\left(1+\gamma_{l}\right)\right]$$

where $W_{l}$ is the spectrum bandwidth allocated to UE *l*. Specifically, when $l\in \left\{pm,pp\right\}$, $W_{l}=\theta W$ and when $l\in \left\{um,up\right\}$, $W_{l}=\left(1-\theta \right)W$. $N_{l}$ is the mean number of serving UEs with user type *l* in a Voronoi cell and its expectation is ENl=AlλuAlλuλlλl+1. If $l\in \left\{pm,um\right\}$, $\lambda_{l}=\lambda_{m}$, otherwise $\lambda_{l}=\lambda_{p}$.

According to the analysis above, we get Lemma 3 as follow:

**Lemma** **3.** *The average achievable downlink rate of a typical UE l can be further represented by*

(9) $$R_{l}=\frac{2\pi \lambda_{l}W_{l}}{A_{l}N_{l}}\int_{0}^{\infty }\int_{0}^{\infty }\exp\left(-\varphi_{l}-\pi r^{2}C_{l}\right)f_{l}\left(r\right)drdt$$

*where $\tau =2^{t}-1$, $\varphi_{l}=-\tau \sigma^{2}r_{l}^{\alpha }\rho_{l}^{-1}P_{l}^{-1}$,*

$$C_{l}=\left\{\begin{array}{c} \lambda_{m}Z\left(\tau ,\alpha ,1\right)+\lambda_{p}{\left({\hat{P}}_{p}/\rho \right)}^{2/\alpha }Z\left(\tau ,\alpha ,B_{p}\right),\mathrm{when}\;l=pm\\ \lambda_{m}{\left(\rho {\hat{P}}_{m}\right)}^{2/\alpha }Z\left(\tau ,\alpha ,{B_{p}}^{-1}\rho^{-1}\right)+\lambda_{p}Z\left(\tau ,\alpha ,1\right),\mathrm{when}\;l=pp\\ \lambda_{m}Z\left(\tau ,\alpha ,1\right)+\lambda_{p}{\left({\hat{P}}_{p}\right)}^{2/\alpha }Z\left(\tau ,\alpha ,B_{p}\right),\mathrm{when}\;l=um\\ \lambda_{m}{\left({\hat{P}}_{m}\right)}^{2/\alpha }Z\left(\tau ,\alpha ,1\right)+\lambda_{p}Z\left(\tau ,\alpha ,1\right),\mathrm{when}\;l=up \end{array}\right.,$$

*where Zτ,α,β=τ22αα∫ββττ22αα∞11+xαα22dx, ${\hat{P}}_{m}=P_{m}/P_{p}$ and ${\hat{P}}_{p}=P_{p}/P_{m}$.*

**Proof.**  The proof is presented in Appendix C. □

**Corollary** **1.** *To further simplify the analysis, we ignore the noise, i.e., $\sigma^{2}=0$ and set the large-scale path loss exponent α=4. In that case, corresponding to the user type, the average achievable downlink rate of four user types can be expressed as:*

(10) $$\begin{array}{c} R_{pm}=\int_{0}^{\infty }\frac{\theta W/A_{pm}N_{pm}}{\lambda_{p,m}Q\left({\hat{P}}_{p}\tau /\rho ,B_{p}{\hat{P}}_{p}/\rho \right)+Q\left(\tau ,1\right)}dt\\ R_{pp}=\int_{0}^{\infty }\frac{\theta W/A_{pp}N_{pp}}{\lambda_{p,m}^{-1}Q\left(\rho {\hat{P}}_{m}\tau ,{B_{p}}^{-1}{\hat{P}}_{m}\right)+Q\left(\tau ,1\right)}-\frac{\theta W/A_{pp}N_{pp}}{\lambda_{p,m}^{-1}\left[Q\left(\rho {\hat{P}}_{m}\tau ,{B_{p}}^{-1}{\hat{P}}_{m}\right)-k_{e}^{-2}+k_{p}^{-2}\right]+Q\left(\tau ,1\right)}dt\\ R_{um}=\int_{0}^{\infty }\frac{\left(1-\theta \right)W/A_{um}N_{um}}{\lambda_{p,m}Q\left({\hat{P}}_{p}\tau ,B_{p}{\hat{P}}_{p}\right)+Q\left(\tau ,1\right)}-\frac{\left(1-\theta \right)W/A_{um}N_{um}}{\lambda_{p,m}\left[Q\left({\hat{P}}_{p}\tau ,B_{p}{\hat{P}}_{p}\right)-k_{e}^{2}+k_{c}^{2}\right]+Q\left(\tau ,1\right)}dt\\ R_{up}=\int_{0}^{\infty }\frac{\left(1-\theta \right)W/A_{up}N_{up}}{\lambda_{p,m}^{-1}Q\left({\hat{P}}_{m}\tau ,{\hat{P}}_{m}\right)+Q\left(\tau ,1\right)}dt, \end{array}$$

*where Qτ,x=x+τarctanττxx, $\lambda_{p,m}=\lambda_{p}/\lambda_{m}$, ${\hat{P}}_{p}=P_{p}/P_{m}$, ${\hat{P}}_{m}=P_{m}/P_{p}$.*

**Proof.**  Set α=4 and $\sigma^{2}=0$, then we can get $\varphi_{l}=0$ and Zτ,α,β=τ∫ββττ∞11+x2dx=τarctanττββ. Combining with Equation (6), the desired results in Equation (9) can be obtained. □

### 4.3. Network Power Consumption

Generally, the BS power consumption comprises static power consumption and transmit power consumption [35]. The static power consumption is caused by signal processing, battery backup, as well as site cooling, and independent with the BS transmit power consumption. The transmit power consumption is determined by the transmission power of this BS and the load-dependent power consumption coefficient of this BS which is denoted as the number of its serving UEs. Define $P_{m,s}$ and $P_{p,s}$ are the static power consumption of each MBS and each PBS, respectively. With FeICIC scheme, the power consumptions of each MBS in PSF and USF can be expressed as $P_{m}^{PFS}=P_{m,s}+N_{pm}\rho P_{m}$ and $P_{m}^{UFS}=P_{m,s}+N_{um}P_{m}$, respectively. Similarly, the power consumptions of each PBS in PSF and USF can be given as $P_{p}^{PFS}=P_{p,s}+N_{pp}P_{p}$ and $P_{p}^{UFS}=P_{p,s}+N_{up}P_{p}$, respectively.

In PFS, the unit area mean power consumption $P^{PFS}$ can be expressed as:

(11) $$P^{PFS}=\lambda_{m}P_{m}^{PFS}+\lambda_{p}P_{p}^{PFS}=\lambda_{m}P_{m,s}+\lambda_{p}P_{p,s}+A_{pm}\lambda_{u}\rho P_{m}+A_{pp}\lambda_{u}P_{p}.$$

Similarly, the unit area mean power consumption in UFS $P^{UFS}$ can be obtained as follow:

(12) $$P^{UFS}=\lambda_{m}P_{m}^{UFS}+\lambda_{p}P_{p}^{UFS}=\lambda_{m}P_{m,s}+\lambda_{p}P_{p,s}+A_{um}\lambda_{u}P_{m}+A_{up}\lambda_{u}P_{p}.$$

Hence, the network power consumption can be expressed as:

(13) $$\begin{array}{c} P^{total}=\theta P_{PFS}+\left(1-\theta \right)P_{UFS}\\ \;\;\;\;\;\;\;\;\;=\lambda_{m}P_{m,s}+\theta \left(A_{pm}\lambda_{u}\rho P_{m}+A_{pp}\lambda_{u}P_{p}\right)+\lambda_{p}P_{p,s}+\left(1-\theta \right)\left(A_{um}\lambda_{u}P_{m}+A_{up}\lambda_{u}P_{p}\right). \end{array}$$

### 4.4. Network Energy Efficiency

The network EE can be defined as the ratio of the achievable network throughput to the network power consumption [37]. For the convenience of derivation, we set $\sigma^{2}\;=\;0$ and α=4. Based on Equations (10) and (13), we can get the closed-form expression of network EE in the following:

(14) $$\begin{array}{c} EE=\frac{R^{total}}{P^{total}}=\frac{\lambda_{u}\left(R_{pm}A_{pm}+R_{pp}A_{pp}+R_{um}A_{um}+R_{up}A_{up}\right)}{P^{total}}\\ \;\;\;\;\;\;\;\;\;\;\;\;\;\;=\frac{\lambda_{u}}{P^{total}}\int_{0}^{\infty }\frac{\theta W/N_{pm}}{\lambda_{p,m}Q\left({\hat{P}}_{p}\tau /\rho ,B_{p}{\hat{P}}_{p}/\rho \right)+Q\left(\tau ,1\right)}\\ \;\;\;\;\;\;\;\;\;\;\;\;\;\;+\frac{\theta W/N_{pp}}{\lambda_{p,m}^{-1}Q\left(\rho {\hat{P}}_{m}\tau ,{B_{p}}^{-1}{\hat{P}}_{m}\right)+Q\left(\tau ,1\right)}-\frac{\theta W/N_{pp}}{\lambda_{p,m}^{-1}\left[Q\left(\rho {\hat{P}}_{m}\tau ,{B_{p}}^{-1}{\hat{P}}_{m}\right)-k_{e}^{-2}+k_{p}^{-2}\right]+Q\left(\tau ,1\right)}\\ \;\;\;\;\;\;\;\;\;\;\;\;\;\;+\frac{\left(1-\theta \right)W/N_{um}}{\lambda_{p,m}Q\left({\hat{P}}_{p}\tau ,B_{p}{\hat{P}}_{p}\right)+Q\left(\tau ,1\right)}-\frac{\left(1-\theta \right)W/N_{um}}{\lambda_{p,m}\left[Q\left({\hat{P}}_{p}\tau ,B_{p}{\hat{P}}_{p}\right)-k_{e}^{2}+k_{c}^{2}\right]+Q\left(\tau ,1\right)}\\ \;\;\;\;\;\;\;\;\;\;\;\;\;\;+\frac{\left(1-\theta \right)W/N_{up}}{\lambda_{p,m}^{-1}Q\left({\hat{P}}_{m}\tau ,{\hat{P}}_{m}\right)+Q\left(\tau ,1\right)}dt\;\;\;\;\;\;\;\; \end{array}$$

## 5. Joint Optimization of FeICIC Parameters and Base-Station Density

Due to the fact that MBSs are usually deployed by network operators, MBS density will change slowly and can be assumed to be constant for analysis simplification. Further simplification of the problem analysis, MBS transmit power in USF and PBS transmit power can also be assumed to be constant without considering power control. Moreover, the PSF ratio can be calculated according to Equation (7). Hence, the network EE is mainly impacted by pico CRE bias, power reduction factor, and PBS density under different UE density, i.e., network load. As MBS density is a constant value, we can first obtain the optimal value of the ratio between PBS density and MBS density $\lambda_{p,m}$, denoted as $\lambda_{p,m}^{*}$, to maximize the network EE. Then the optimal PBS density $\lambda_{p}^{*}$ can be calculated by $\lambda_{p}^{*}=\lambda_{m}\lambda_{p,m}^{*}$. Thus, the joint optimization problem with the object of network EE maximization can be formulated as follows:

(15) $$\begin{array}{c} \underset{\rho ,B_{p},\lambda_{p,m}}{\arg\;\max}EE\\ s.t.\;0<B_{p}\leq 25\;\mathrm{dB}\\ \;\;\;\;\;\;0\leq \rho <1\\ \;\;\;\;\;\;0<\lambda_{p,m}\leq 30 \end{array}$$

However, the network EE is nonlinear with $\lambda_{p,m}$, $B_{p}$ and ρ, which is difficult to obtain the optimal $\lambda_{p,m}$, $B_{p}$ and ρ at the same time with reasonable complexity. Note that the value ranges of $\lambda_{p,m}$, $B_{p}$ and ρ are limited, which make it possible to seek out the optimal values of $\lambda_{p,m}$, $B_{p}$ and ρ through a linear search algorithm by fixing two of these three variables, respectively. Therefore, we propose a heuristic algorithm to obtain the sub-optimal solution of the joint optimization problem in Equation (15). The proposed heuristic algorithm decomposes the original optimization problem into two sub-problems including FeICIC parameters optimization and PBS density optimization. For FeICIC parameters optimization, we first derive the approximate relation between $B_{p}$ and ρ. Then, we get the sub-optimal values of $B_{p}$ and ρ with given $\lambda_{p,m}$ by an alternating algorithm. For PBS density optimization, the optimal value of $\lambda_{p,m}$ can be obtained by a linear search method based on fixed $B_{p}$ and ρ. Finally, we alternately solve two sub-problems to achieve globally optimal values of these variables.

### 5.1. Joint Optimization of Pico CRE Bias and Power Reduction Factor

In order to get the optimal values of $B_{p}$ and ρ for network EE maximization, suppose that $\lambda_{p,m}$ and $\lambda_{u}$ are given. Thus, the pico CRE bias and power reduction factor joint optimization problem can be formulated as follow:

(16) $$\begin{array}{c} \rho^{*},B_{p}^{*}=\underset{\rho ,B_{p}}{\arg\;\max}EE|\lambda_{p,m}\\ s.t.\;0<B_{p}\leq 25\;\mathrm{dB}\\ \;\;\;\;\;\;0\leq \rho <1\\ \;\;\;\;\;\;\lambda_{p,m}\;\mathrm{is}\;\mathrm{an}\;\mathrm{arbitrary}\;\mathrm{constant}\;\mathrm{between}\;0\;\mathrm{and}\;30, \end{array}$$

where $B_{p}^{*}$ and $\rho^{*}$ are the optimal values of pico CRE bias and power reduction factor, respectively. To simplify the solving process, we assume that the overall SINR of PSF MUEs is identical to that of the USF MUEs. Hence, the result of resource allocation will have a direct influence on the network *EE*. In view of user fairness, the optimal network EE can be achieved when the relationship of association probabilities of PSF MUEs and USF MUEs obey the Equation (17).

(17) $$\theta =\frac{A_{pm}}{A_{pm}+A_{um}}.$$

**Figure 5:**
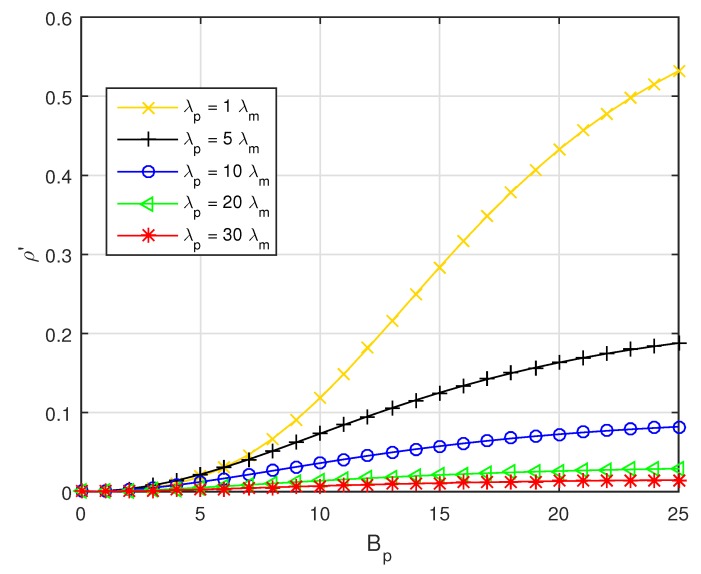
The near-optimal power reduction factor $\rho^{\prime }$ versus $B_{p}$ with fixed $\lambda_{u}=0.0018$.

Combining with Equation (7), we can get the approximate relation of ρ and $B_{p}$ as:

(18) $$\rho^{\prime }=\frac{B_{p}P_{p}}{P_{m}{\left(k_{e}^{2}/A_{pp}-\lambda_{m}/\lambda_{p}\right)}^{2}},$$

where $\rho^{\prime }$ denotes the approximate near-optimal value of ρ. The relationships between ρ and $B_{p}$ under different $\lambda_{p}$ are shown in Figure 5. We can find that $\rho^{\prime }$ is a strictly increasing function with respect to $B_{p}$ at a given PBS density.

Substituting $\rho^{\prime }$ into Equation (14), we can get the near-optimal value of $B_{p}$, denoted as ${\hat{B}}_{p}^{*}$ by solving the following univariate problem.

(19) $$\begin{array}{c} {\hat{B}}_{p}^{*}=\underset{B_{p}}{\arg\;\max}EE|\lambda_{p,m},\rho =\rho^{\prime }\\ s.t.\;0<B_{p}\leq 25\;\mathrm{dB}\\ \;\;\;\;\;\;\lambda_{p,m}\;\mathrm{is}\;\mathrm{an}\;\mathrm{arbitrary}\;\mathrm{constant}\;\mathrm{between}\;0\;\mathrm{and}\;30 \end{array}$$

Then, combining the value of ${\hat{B}}_{p}^{*}$ with Equation (14), the near-optimal value of ρ, denoted as ${\hat{\rho }}^{*}$ can be obtained by following univariate problem.

(20) $$\begin{array}{c} {\hat{\rho }}^{*}=\underset{\rho }{\arg\;\max}EE|\lambda_{p,m},{\hat{B}}_{p}^{*}\\ s.t.\;0\leq \rho <1\\ \;\;\;\;\;\;\lambda_{p,m}\;\mathrm{is}\;\mathrm{an}\;\mathrm{arbitrary}\;\mathrm{constant}\;\mathrm{between}\;0\;\mathrm{and}\;30 \end{array}$$

Thus, ${\hat{B}}_{p}^{*}$ and ${\hat{\rho }}^{*}$ are obtained by an alternating algorithm, which is shown in Algorithm 1. $\Delta_{1}$ and $\Delta_{2}$ represent the step lengths of $B_{p}$ and ρ, which are set to be 0.1 and 0.05, respectively. $EE^{*}$ denotes the optimal value of the network EE. The main idea of this altering algorithm is to obtain the near-optimal values of pico CRE bias and power reduction factor by a two-step linear search approach on the basis of the approximate relationship between them. In line 1 of Algorithm 1, the network scenario and relative parameters are initialized. From line 2 to line 10, the optimal pico CRE bias is obtained by a linear search way on the basis of approximate relationship between pico CRE bias and power reduction factor. From line 11 to line 18, the optimal power reduction factor is calculated based on Equation (14).

 **Algorithm 1:** Joint pico CRE bias and power reduction factor optimization (JBPO) algorithm.
   1:**Initialization**: Initialize the network scenario and the values $\lambda_{u}$, $\lambda_{m}$ and $\lambda_{p,m}$, where $\lambda_{p,m}\in \left(0,30\right]$. Set $\Delta_{1}=0.5\;\mathrm{dB}$, $\Delta_{2}=0.05$, ${\hat{B}}_{p}^{*}=B_{p}=0.5\;\mathrm{dB}$ and $EE^{*}=0$.   2:
**while** $B_{p}\leq 25\;\mathrm{dB}$ **do**
   3: Substituting $B_{p}$ into Equation (18), the approximate near-optimal value of the power reduction factor ρ can be obtained as $\rho^{\prime }$.   4: $\rho =\rho^{\prime }$.   5: Substituting $B_{p}$ and ρ into Equation (14) with given $\lambda_{u}$, $\lambda_{m}$ and $\lambda_{p,m}$, the current network energy efficiency $EE^{\prime }$ can be obtained as: $EE^{\prime }=EE\left(B_{p},\rho \right)|\lambda_{u},\lambda_{m},\lambda_{p,m}$.   6: **if** $EE^{\prime }>EE^{*}$ **then**   7:  ${\hat{B}}_{p}^{*}=B_{p}$, $EE^{*}=EE^{\prime }$.   8: **end if**    9: $B_{p}=B_{p}+\Delta_{1}$.  10:**end while**   11:${\hat{\rho }}^{*}=\rho =0$, $B_{p}={\hat{B}}_{p}^{*}$.  12:
**while** ρ<1 **do**
  13: Substituting ρ into Equation (14) with given $\lambda_{u}$, $\lambda_{m}$, $\lambda_{p,m}$ and $B_{p}$, the current network energy efficiency $EE^{\prime }$ can be obtained as: $EE^{\prime }=EE\left(\rho \right)|\lambda_{u},\lambda_{m},\lambda_{p,m},B_{p}$.  14: **if** $EE^{\prime }>EE^{*}$ **then**
  15:  ${\hat{\rho }}^{*}=\rho$, $EE^{*}=EE^{\prime }$.  16: **end if**   17: $\rho =\rho +\Delta_{2}$.  18:
**end while**



### 5.2. Optimization of PBS Density

Similarly, suppose that $B_{p}$, ρ and $\lambda_{u}$ are known. Thus, the PBS density optimization problem can be formulated as follow:

(21) $$\begin{array}{c} \lambda_{p,m}^{*}=\underset{\lambda_{p,m}}{\arg\;\max}EE|\rho ,B_{p}\\ s.t.\;0<\lambda_{p,m}\leq 30\\ \;\;\;\;B_{p}\;\mathrm{is}\;\mathrm{an}\;\mathrm{arbitrary}\;\mathrm{constant}\;\mathrm{between}\;0\;\mathrm{and}\;25\\ \;\;\;\;\rho \;\mathrm{is}\;\mathrm{an}\;\mathrm{arbitrary}\;\mathrm{constant}\;\mathrm{between}\;0\;\mathrm{and}\;1 \end{array}$$

Assume that the step length of $\lambda_{p,m}$ is $\Delta_{3}$, which is set to be 0.03. Thus, the optimal PBS density can be obtained by a linear search algorithm to maximize the network EE, which is described in Algorithm 2. Line 1 of Algorithm 2 indicates the network scenario and parameters initialization. From line 2 to line 8, the optimal PBS density is acquired by a linear search method to maximize the network *EE*.    

 **Algorithm 2:** Pico base stations (PBS) density optimization (PDO) algorithm.
 1:**Initialization**: Initialize the network scenario and the values $\lambda_{u}$, $\lambda_{m}$, $B_{p}$ and ρ, where $B_{p}\in \left(0,25\right]$ and $\rho \in \left[0,1\right)$. Set $\Delta_{3}=0.03$, $\lambda_{p,m}=0.3$, $\lambda_{p}^{*}=\lambda_{p,m}\lambda_{m}$ and $EE^{*}=0$. 2:
**while** $\lambda_{p,m}\leq 30$ **do**
 3: Substituting $\lambda_{p,m}$ into Equation (14) with given $\lambda_{u}$, $\lambda_{m}$, $B_{p}$ and ρ, the current network energy efficiency $EE^{\prime }$ can be obtained as: $EE^{\prime }=EE\left(\lambda_{p,m}\right)|\lambda_{u},\lambda_{m},B_{p},\rho$. 4: **if** $EE^{\prime }>EE^{*}$ **then**
 5:  $\lambda_{p}^{*}=\lambda_{p,m}\lambda_{m}$, $EE^{*}=EE^{\prime }$. 6: **end if**  7: $\lambda_{p,m}=\lambda_{p,m}+\Delta_{3}$. 8:
**end while**



### 5.3. Joint Optimization of Pico CRE Bias, Power Reduction Factor and PBS Density

The FeICIC parameter optimization sub-problem and the PBS density optimization sub-problem are solved independently by the aforementioned optimization algorithms. Due to the fact that these variables are affected by each other, we further propose a heuristic pico CRE bias, power reduction factor and PBS density joint optimization algorithm to globally optimize network EE based on the joint pico CRE bias and power reduction factor optimization (JBPO) algorithm and PDO algorithm. The detailed procedure of our proposed heuristic algorithm is summarized in Algorithm 3. ε represents the positive tolerance value. $N_{loop}$ is the iteration times of the algorithm. Line 1 of Algorithm 3 signifies the network scenario and parameters initialization. From line 3 to line 4, the JBPO algorithm is executed to obtain the current optimal pico CRE bias and power reduction factor at a given PBS density. From line 5 to line 6, the PDO algorithm is performed to acquire the current optimal PBS density based on the above obtained pico CRE bias and power reduction factor. From line 2 to line 9, the network EE is iteratively optimized until it cannot improve further within an arbitrary value ε. As a result, the optimal pico CRE bias, power reduction factor and PBS density are obtained and the network EE is maximized.

 **Algorithm 3:** Joint pico pico-cell range expansion (CRE) bias, power reduction factor and PBS density optimization (JBPDO) algorithm.
 1:**Initialization**: Initialize the network scenario and the values $\lambda_{u}$, $\lambda_{m}$, $B_{p}$, ρ and $\lambda_{p,m}$, where $B_{p}\in \left(0,25\right]$, $\rho \in \left[0,1\right)$ and $\lambda_{p,m}\in \left(0,30\right]$. Set $EE^{*}=0$, $N_{loop}=0$ and ε>0. 2:
**repeat**
 3: Solving the optimization problem in Equation (16), the near-optimal pico CRE bias ${\hat{B}}_{p}^{*}$ and the near-optimal power reduction factor ${\hat{\rho }}^{*}$ at given $\lambda_{p,m}$ can be obtained based on the JBPO algorithm.   4: $B_{p}={\hat{B}}_{p}^{*}$, $\rho ={\hat{\rho }}^{*}$. 5: Solving the optimization problem in Equation (21), the near-optimal PBS density ${\hat{\lambda }}_{p}^{*}$ at given $B_{p}$ and ρ can be obtained based on the PDO algorithm.   6: $\lambda_{p}={\hat{\lambda }}_{p}^{*}$, $\lambda_{p,m}={\hat{\lambda }}_{p}^{*}/\lambda_{m}$. 7: Substituting $B_{p}$, ρ and $\lambda_{p,m}$ into Equation (14) with given $\lambda_{u}$ and $\lambda_{m}$, the current network energy efficiency $EE^{\prime }$ can be obtained as: $EE^{\prime }=EE\left(B_{p},\rho ,\lambda_{p,m}\right)|\lambda_{u},\lambda_{m}$. 8: $B_{p}^{*}=B_{p}$, $\rho^{*}=\rho$, $\lambda_{p}^{*}=\lambda_{p}$, $EE^{*}=EE^{\prime }$, $N_{loop}=N_{loop}+1$. 9:
**until** $\left|EE^{\prime }-EE^{*}\right|<\varepsilon$



### 5.4. Computational Complexity

The computational complexity of the JBPO algorithm can be calculated as $O\left(n_{B_{p}}+n_{\rho }\right)$, where $n_{B_{p}}$ and $n_{\rho }$ are the space sizes of the linear search for $B_{p}$ and ρ, respectively. The computational complexity of the PDO algorithm is $O\left(n_{\lambda_{p}}\right)$, where $n_{\lambda_{p}}$ is the space size of the linear search for $\lambda_{p}$. Thus, the computational complexity of the JBPDO algorithm is $O\left[\left(n_{B_{p}}+n_{\rho }+n_{\lambda_{p}}\right)\times N_{loop}\right]$.

Due to the fact that there does not an exist effective algorithm for solving Equation (15), we compare the computational complexity of our proposed JBPDO algorithm with that of a traversal algorithm. As for solving Equation (15), the optimal values of pico CRE bias, power reduction factor and PBS density can be obtained by a traversal way, i.e., traversal pico CRE bias, power reduction factor and PBS density optimization (TBPDO) algorithm, which refers to traversing all possible values of these three parameters to maximize the objective function in Equation (15). Thus, the computational complexity of TBPDO algorithm will be $O\left(n_{B_{p}}\times n_{\rho }\times n_{\lambda_{p}}\right)$, which signifies that the computational complexity of our proposed JBPDO algorithm is reduced effectively.

As for solving the objective function in Equation (16), the optimal values of pico CRE bias and power reduction factor can also be obtained by a traversal way, i.e., traversal pico CRE bias and power reduction factor optimization (TBPO) algorithm, which refers to traversing all possible values of these two parameters to maximize the objective function in Equation (16). Indeed, TBPO algorithm consists of two nested ergodic sub-processes: (1) traversal pico CRE bias optimization (TBO) algorithm, which is executed by traversing all pico CRE bias to maximize the objective function in Equation (19) under fixed power reduction factor and PBS density; (2) traversal power reduction factor optimization (TPO) algorithm, which is executed by traversing all power reduction factor to maximize the objective function in Equation (20) under fixed pico CRE bias and PBS density. Therefore, the computational complexities of the TBO and TPO algorithms are $O\left(n_{B_{p}}\right)$ and $O\left(n_{\rho }\right)$, respectively. As a result, the computational complexity of the TBPO algorithm will be $O\left(n_{B_{p}}\times n_{\rho }\right)$, which is obviously higher than that of our proposed JBPO algorithm.

## 6. Numerical Results and Analysis

In this section, we not only provide theoretical simulation, but also verify the effectiveness of proposed heuristic algorithms by Monte Carlo simulation. In the theoretical simulation, we considerws a network coverage area within a square region of 1000m×1000m. The deployments of PBS and MBS follow the PPP model and the typical UE is deployed in the origin. The simulation parameters used in this paper are summarized in Table 2. We took the average results from 30 times of network implementations as Monte Carlo simulations results. In each network implementation, the locations of MBSs, PBSs and UEs were modeled as spatial PPP, respectively. Then, the network EEs was calculated for all the different combination of pico CRE bias, power reduction factor, and PBS density values within their value ranges based on wireless channel quality. Finally, the maximum network EE and the optimal pico CRE bias, power reduction factor and PBS density can be obtained by comparing the calculated network EEs under all combinations. Indeed, the results of Monte Carlo simulation, including the maximal network EE, the optimal pico CRE bias, the optimal power reduction factor and the optimal PBS density, were obtained by a traversal way in each network implementation. Meanwhile, the performances of algorithms shown in the following simulation figures were just simulated based on theoretical derived Equation (14). Therefore, Monte Carlo simulation results had the best performance and can be referred to as a baseline for valuing the performances of those algorithms.

**Figure 6:**
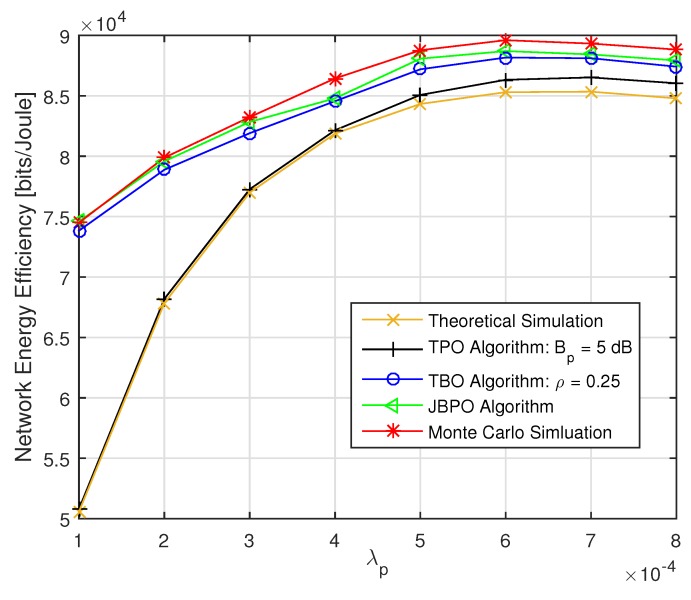
The network energy efficiency (EE) versus $\lambda_{p}$ with fixed $\lambda_{u}=0.0018$.

**Figure 7:**
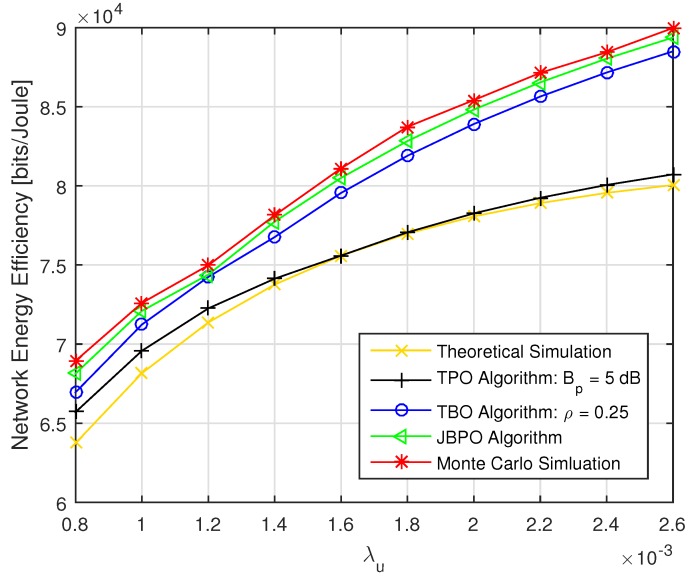
The network EE versus $\lambda_{u}$ with fixed $\lambda_{p}=10\lambda_{m}$.

At first, the network EE performances of the JBPO algorithm were compared with those of the TPO algorithm, TBO algorithm, theoretical simulation and Monte Carlo simulation, as shown in Figure 6 and Figure 7, respectively. As shown in Figure 6, with the increase of PBS density, network EE increased accordingly. With PBS density increase, more UEs were offloaded into the coverage of low power PBSs. Then the distance between the transmitter and the receiver was shorted, resulting in network EE improvement. As shown in Figure 7, with the increase of UE density, the curves of network EE also rose accordingly. As the UE density increased, more UEs can be offloaded into the coverage of low power PBSs by adjusting pico CRE bias and power reduction factor via network EE optimization algorithms. Then network EE can be improved. For the theoretical simulation curve, because the network EE was not optimized and just calculated according to Equation (14) with pico CRE bias and power reduction factor being set to be fixed values 5 dB and 0.25, respectively, so it had the worst performance. The TPO algorithm and the TBO algorithm optimized power reduction factor and CRE bias, respectively. Therefore, the performances of these two algorithms with one parameter optimized were better than that of the theoretical simulation. Our proposed JBPO algorithm can jointly optimize pico CRE bias and power reduction factor together. Therefore, it can further improve the network EE and match the Monte Carlo simulation results very well. Furthermore, the performance of the TPO algorithm was far less than that of the TBO algorithm, which signifies that the influence of pico CRE bias was more than that of the power reduction factor on the network EE, especially in low PBS density. In addition, in Figure 7 the performance gap between the JBPO algorithm and theoretical simulation increases accordingly with the growth of UE density, which further indicates the importance of joint pico CRE bias and power reduction factor optimization for the heavy network load scenario.

**Figure 8:**
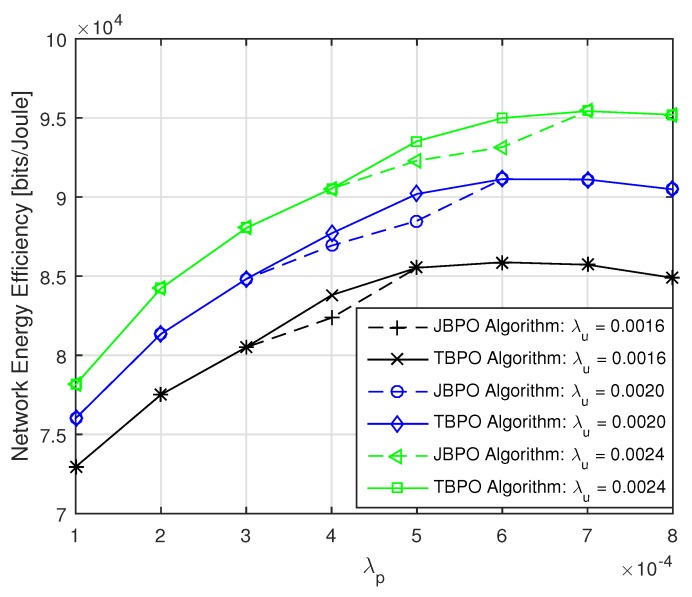
The network EE versus $\lambda_{p}$ with different $\lambda_{u}$.

**Figure 9:**
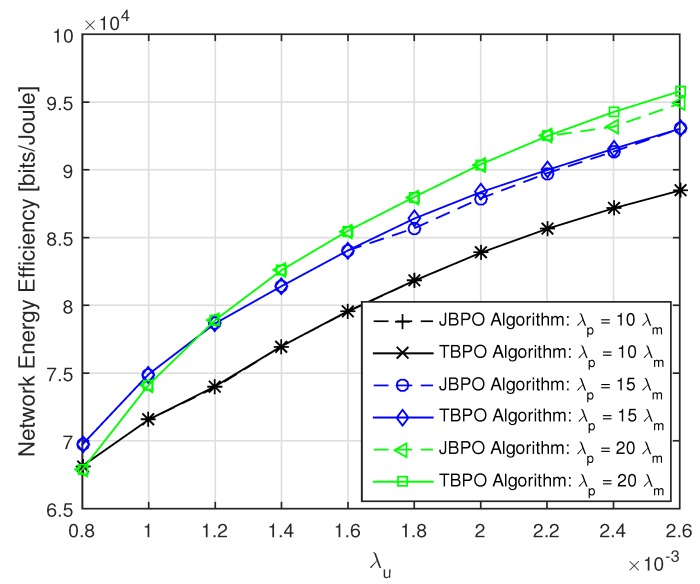
The network EE versus $\lambda_{u}$ with different $\lambda_{p}$.

Then, the performances of our proposed JBPO algorithm were compared with that of the TBPO algorithm in Figure 8 and Figure 9 from different aspects, respectively. The relationship between network EE and $\lambda_{p}$ with different $\lambda_{u}$ are shown in Figure 8. We can see that the performance of our proposed JBPO algorithm was just slightly worse than that of the TBPO algorithm, but the computational complexity of JBPO was much lower than that of the TBPO algorithm. In addition, all curves of network EE increased first and then fell down slightly with PBS density increase, which illustrates that increasing PBS density can improve the network EE significantly within a certain network load. Nonetheless, when the PBS density exceeded a certain limit, further increasing will cause more complex interference and more power consumption, which will result in the network EE deterioration.

The relationship between network EE and $\lambda_{u}$ with different $\lambda_{p}$ are depicted in Figure 9. Due to the curves cross with each other under different UE densities, the PBS density should be carefully adjusted according to the network load fluctuation. In addition, for a given PBS density, we can see that the network EE become greater with a higher network load. Referring to Figure 8 and Figure 9 together, although the network EE performance of the TBPO algorithm is just slightly better than that of the JBPO algorithm, the computational complexity of it is $O(n_{B_{p}}\times n_{\rho })$, which is much higher than that of the JBPO algorithm.

**Figure 10:**
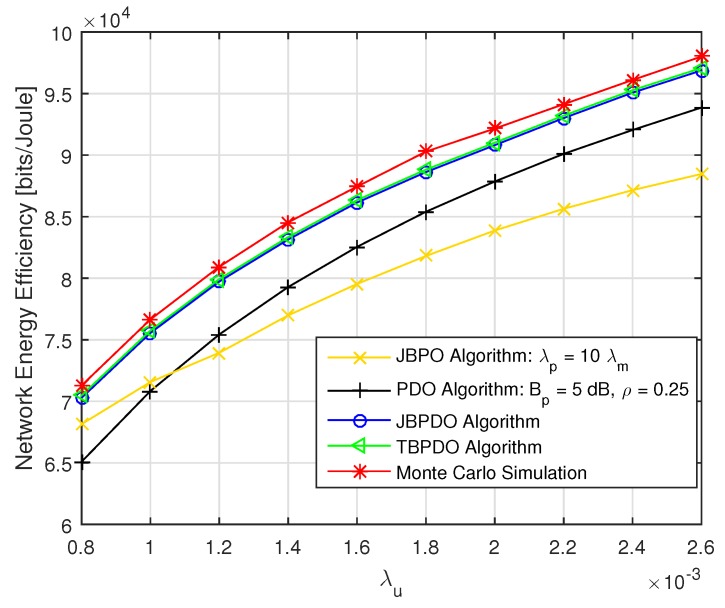
The network EE versus $\lambda_{u}$ with different optimization algorithms.

Finally, the network EE performances of the JBPDO algorithm were compared with those of the PDO algorithm, JBPO algorithm with fixed $\lambda_{p}=10\lambda_{m}$, TBPDO algorithm, and Monte Carlo simulation in Figure 10. In the TBPDO algorithm, pico CRE bias, power reduction factor and PBS density are jointly optimized to maximize network EE by an exhaustive traversal algorithm based on Equation (14). The Monte Carlo simulation results show the maximum network EE within the value range of pico CRE bias, power reduction factor and PBS density at different network load.

**Figure 11:**
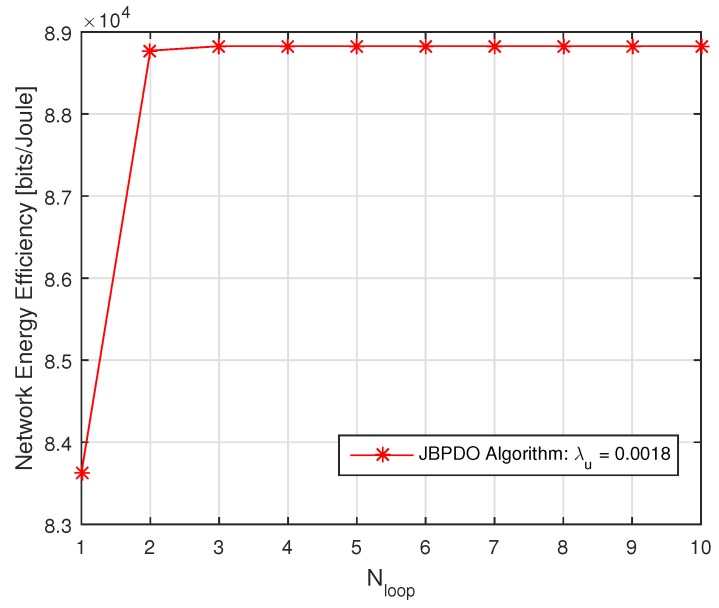
The network EE versus $N_{loop}$ for JBPDO algorithm.

As shown in Figure 10, the proposed JBPDO algorithm can obtain better network EE than that of the JBPO algorithm since the JBPDO algorithm further optimizes the PBS density on the basis of the JBPO algorithm. Meanwhile, the accuracy and effectiveness of our proposed JBPDO algorithm are once again verified by Monte Carlo simulation results. In addition, although the network EE of the TBPDO algorithm outperforms our proposed JBPDO algorithm, the computational complexity of TBPDO algorithm is $O\left(n_{B_{p}}\times n_{\rho }\times n_{\lambda_{p}}\right)$, which is far higher than that of the JBPDO algorithm. The convergence of JBPDO algorithm is provided in Figure 11. From Figure 11, we can find that JBPDO algorithm can converge after three iterations. It is proved that the computational complexity of the JBPDO algorithm is much lower than that of the TBPDO algorithm and more suitable for the real-time network.

## 7. Conclusions

In this paper, pico CRE bias, PSF power reduction factor and PBS density are jointly optimized to maximize network EE for a two-tier HetNets with FeICIC. First, we derive the closed-form expression of network EE based on stochastic geometry theory. Then, the near-optimal values of pico CRE bias and power reduction factor are obtained by an alternating algorithm based on the equivalence relation between them at a given PBS density deployment. With fixed pico CRE bias and power reduction factor, the PBS density is optimized by a linear search method. Finally, a heuristic algorithm is proposed to optimize the pico CRE bias, power reduction factor, and PBS density jointly for network EE maximization. Extensive simulation results show the accuracy of network EE theoretical deduction and the effectiveness of our proposed low-complexity heuristic algorithm for network EE improvement.

## Figures and Tables

**Table 1:** Notations summary.

Notation	Description
$\lambda_{m}$, $\lambda_{p}$, $\lambda_{u}$	Density of MBS, PBS and UE
L, *l*	Set of user types, indication of the user type
*W*	Total spectrum bandwidth
*h*	Channel fast fading gain
α	Large-scale path loss exponent
$\sigma^{2}$	Thermal noise
$P_{m}$, $P_{p}$	Maximum transmission power of MBS and PBS
ρ	Power reduction factor
θ	PSF ratio
$B_{p}$	Pico CRE bias
$\gamma_{l}$	SINR of a typical UE *l*
$I_{m}$, $I_{p}$	Full power aggregate interference from macro tier and pico tier
$r_{m}$, $r_{p}$	Distance from a UE to its nearest MBS and its nearest PBS
$A_{l}$	Probability of a typical UE belongs to the user type *l*
$k_{c}$, $k_{e}$, $k_{p}$	Factor of macro-cell center region, pico CRE region and pico-cell original coverage region
$r_{l}$	Distance from a typical UE *l* and its serving BS
$f_{l}\left(r\right)$	PDF of the distance between a UE and its serving BS
$\lambda_{l}$	Density of BSs associated with user type *l*
$R_{l}$	Mean achievable downlink data rate of a typical UE *l*
$W_{l}$	Spectrum bandwidth allocated to a typical UE *l*
$N_{l}$, $N_{l}^{total}$	Mean number of UEs with user type *l* in a Voronoi cell, total number of UEs with user type *l*
$P_{m,s}$, $P_{p,s}$	Static power of MBS and PBS
${\hat{P}}_{m}$	Proportion between maximum transmission power of MBS and that of PBS
${\hat{P}}_{p}$	Proportion between maximum transmission power of PBS and that of MBS
$\lambda_{p,m}$	Proportion between PBS density and MBS density
$R^{total}$	Total network throughput
$P^{total}$	Total network power consumption
$\rho^{\prime }$	Approximate value of power reduction factor
${\hat{B}}_{p}^{*}$, ${\hat{\rho }}^{*}$, ${\hat{\lambda }}_{p}^{*}$	Near-optimal value of pico CRE bias, power reduction factor and PBS density
$B_{p}^{*}$, $\rho^{*}$, $\lambda_{p}^{*}$, $EE^{*}$	Optimal value of pico CRE bias, power reduction factor, PBS density and EE

**Table 2:** Network scenario parameters.

Parameters	Value
Carrier frequency *f*	2 GHz
Total spectrum bandwidth *W*	10 MHz
Path loss exponent α	4
Path loss *L*	$L=10\log\left(L_{0}\right)+\alpha 10\log\left(r\right)$, where L0=4πf4πfcc2, $c=3\times 10^{8}$ m/s
MBS transmission power $P_{m}$	43 dBm
PBS transmission power $P_{p}$	30 dBm
MBS static power $P_{m,s}$	800 W
PBS static power $P_{p,s}$	130 W
MBS density $\lambda_{m}$	0.00003

## Appendix A. Proof of Lemma 1

Considering a typical UE, it is at a distance $r_{k}$ away from its nearest BS in the *k* tier, that is to say, there is no BS closer than $r_{k}$ in the *k* tier. Due to the locations of MBSs and PBSs follow two iid. PPPs, the cumulative distribution function (CDF) of two-point distance $r_{k}$ is

(A1) $$\begin{array}{c} F_{r_{k}}\left(r\right)=1-\mathrm{Prob}\left(r_{k}>r\right)\\ \;\;\;\;\;\;\;\;\;\;=1-\mathrm{Prob}\left(no\;BS\;closer\;than\;r_{k}\right)\\ \;\;\;\;\;\;\;\;\;\;=1-\exp\left(-\pi r^{2}\lambda_{k}\right), \end{array}$$

where $\lambda_{k}$ is the BS density of *k* tier. We can obtain the PDF of the distance by the differential of Equation (A1) over *r* as follows:

(A2) $$f_{r_{k}}\left(r\right)=2\pi r\lambda_{k}\exp\left(-\pi r^{2}\lambda_{k}\right).$$

Hence, given an arbitrary coefficient value of *n*, the probability of $r_{p}>nr_{m}$ can be given as:

(A3) $$\begin{array}{c} \mathrm{Prob}\left(r_{p}>nr_{m}\right)=\mathrm{Prob}\left(no\;BS\;closer\;than\;nr_{m}|r_{m}\right)\\ \;\;\;\;\;\;\;\;\;\;\;\;\;\;\;\;\;\;\;\;\;\;\;\;\;\;\;=\mathrm{Prob}\left(r_{p}>nr_{m},r_{m}\right)\\ \;\;\;\;\;\;\;\;\;\;\;\;\;\;\;\;\;\;\;\;\;\;\;\;\;\;\;=\int_{0}^{\infty }\mathrm{Prob}\left(r_{p}>nr_{m},r_{m}\right)f_{r_{m}}\left(r\right)dr\\ \;\;\;\;\;\;\;\;\;\;\;\;\;\;\;\;\;\;\;\;\;\;\;\;\;\;\;=\int_{0}^{\infty }\left[\int_{nr}^{\infty }f_{r_{p}}\left(r\right)dr\right]f_{r_{m}}\left(r\right)dr\;\\ \;\;\;\;\;\;\;\;\;\;\;\;\;\;\;\;\;\;\;\;\;\;\;\;\;\;\;=\int_{0}^{\infty }\left[\int_{nr}^{\infty }2\pi r\lambda_{p}\exp\left(-\pi r^{2}\lambda_{p}\right)dr\right]f_{r_{m}}\left(r\right)dr\\ \;\;\;\;\;\;\;\;\;\;\;\;\;\;\;\;\;\;\;\;\;\;\;\;\;\;\;=\int_{0}^{\infty }\left[-\exp\left(-\pi r^{2}n^{2}\lambda_{p}\right)\right]2\pi r\lambda_{m}\exp\left(-\pi r^{2}\lambda_{m}\right)dr\\ \;\;\;\;\;\;\;\;\;\;\;\;\;\;\;\;\;\;\;\;\;\;\;\;\;\;\;=\frac{\lambda_{m}}{\lambda_{m}+n^{2}\lambda_{p}}. \end{array}$$

Further, given two arbitrary coefficient values of $n_{a}$ and $n_{b}$, the probability of $n_{a}r_{m}<r_{p}<n_{b}r_{m}$ can be expressed as:

(A4) $$\begin{array}{c} \mathrm{Prob}\left(n_{a}r_{m}<r_{p}<n_{b}r_{m}\right)=\mathrm{Prob}\left(r_{p}>n_{a}r_{m}\right)-\mathrm{Prob}\left(r_{p}>n_{b}r_{m}\right)\\ \;\;\;\;\;\;\;\;\;\;\;\;\;\;\;\;\;\;\;\;\;\;\;\;\;\;\;\;\;\;\;\;\;\;\;\;\;\;\;\;=\frac{\lambda_{m}}{\lambda_{m}+n_{a}^{2}\lambda_{p}}-\frac{\lambda_{m}}{\lambda_{m}+n_{b}^{2}\lambda_{p}}. \end{array}$$

Combing Equation (A4) with Equation (2), the association probabilities of the typical UE can be obtained as Equation (5).

## Appendix B. Proof of Lemma 2

According to the Bayes rule, we can obtain the conditional probability as

(A5) $$\mathrm{Prob}\left(r_{m}|r_{p}>nr_{m}\right)=\frac{\mathrm{Prob}\left(r_{m}<r,r_{p}>nr_{m}\right)}{\mathrm{Prob}\left(r_{p}>nr_{m}\right)},$$

where $\mathrm{Prob}\left(r_{p}>nr_{m}\right)$ is given in Equation (A3). After taking partial derivation with respect to the variable *r*, the PDF of distance *r* between a typical UE to its serving BS under the condition of $r_{p}>nr_{m}$ is

(A6) $$\begin{array}{c} f_{r_{m}|r_{p}>nr_{m}}\left(r\right)=\frac{d}{dr}\mathrm{Prob}\left(r_{m}|r_{p}>nr_{m}\right)\\ \;\;\;\;\;\;\;\;\;\;\;\;\;\;\;\;\;\;\;\;\;=\frac{1}{\mathrm{Prob}\left(r_{p}>nr_{m}\right)}\frac{d}{dr}\mathrm{Prob}\left(r_{m}<r,r_{p}>nr_{m}\right)\\ \;\;\;\;\;\;\;\;\;\;\;\;\;\;\;\;\;\;\;\;\;=\frac{2\pi \lambda_{m}}{\mathrm{Prob}\left(r_{p}>nr_{m}\right)}\frac{d}{dr}\int_{0}^{r}\mathrm{Prob}\left(r_{p}>nr_{m}|r_{m}=r\right)f_{r_{m}}\left(r\right)dr\\ \;\;\;\;\;\;\;\;\;\;\;\;\;\;\;\;\;\;\;\;\;=\frac{2\pi \lambda_{m}}{\mathrm{Prob}\left(r_{p}>nr_{m}\right)}\frac{d}{dr}\int_{0}^{r}r\exp\left(-\pi r^{2}n^{2}\lambda_{p}\right)\exp\left(\pi r^{2}\lambda_{m}\right)dr\\ \;\;\;\;\;\;\;\;\;\;\;\;\;\;\;\;\;\;\;\;\;=\frac{\frac{\lambda_{m}}{\lambda_{m}+n^{2}\lambda_{p}}}{\mathrm{Prob}\left(r_{p}>nr_{m}\right)}\frac{d}{dr}\left\{1-\exp\left[-\pi r^{2}\left(\lambda_{m}+n^{2}\lambda_{p}\right)\right]\right\}\\ \;\;\;\;\;\;\;\;\;\;\;\;\;\;\;\;\;\;\;\;\;=\frac{2\pi r\lambda_{m}}{\mathrm{Prob}\left(r_{p}>nr_{m}\right)}\exp\left[-\pi r^{2}\left(\lambda_{m}+n^{2}\lambda_{p}\right)\right]. \end{array}$$

Further, we can obtain as:

(A7) $$\begin{array}{c} f_{r_{m}|n_{a}r_{m}<r_{p}<n_{b}r_{m}}\left(r\right)=\frac{d}{dr}\mathrm{Prob}\left(r_{m}|n_{a}r_{m}<r_{p}<n_{b}r_{m}\right)\\ \;\;\;\;\;\;\;\;\;\;\;\;\;\;\;\;\;\;\;\;\;\;\;\;\;\;\;\;\;\;\;=\frac{d}{dr}\frac{\mathrm{Prob}\left(r_{m}<r,n_{a}r_{m}<r_{p}<n_{b}r_{m}\right)}{\mathrm{Prob}\left(n_{a}r_{m}<r_{p}<n_{b}r_{m}\right)}\\ \;\;\;\;\;\;\;\;\;\;\;\;\;\;\;\;\;\;\;\;\;\;\;\;\;\;\;\;\;\;\;=\frac{1}{\mathrm{Prob}\left(n_{a}r_{m}<r_{p}<n_{b}r_{m}\right)}\frac{d}{dr}\left[\mathrm{Prob}\left(r_{m}<r,r_{p}>n_{a}r_{m}\right)\right.\\ \;\;\;\;\;\;\;\;\;\;\;\;\;\;\;\;\;\;\;\;\;\;\;\;\;\;\;\;\;\;\;\left.-\mathrm{Prob}\left(r_{m}<r,r_{p}>n_{b}r_{m}\right)\right]\\ \;\;\;\;\;\;\;\;\;\;\;\;\;\;\;\;\;\;\;\;\;\;\;\;\;\;\;\;\;\;\;=\frac{2\pi r\lambda_{m}}{\mathrm{Prob}\left(n_{a}r_{m}<r_{p}<n_{b}r_{m}\right)}\left\{\exp\left[-\pi r^{2}\left(\lambda_{m}+n_{a}^{2}\lambda_{p}\right)\right]\right.\\ \;\;\;\;\;\;\;\;\;\;\;\;\;\;\;\;\;\;\;\;\;\;\;\;\;\;\;\;\;\;\;\left.-\exp\left[-\pi r^{2}\left(\lambda_{m}+n_{b}^{2}\lambda_{p}\right)\right]\right\}. \end{array}$$

According to Equations (5) and (A7), we can get the PDFs of distance *r* between a typical UE *l* to its serving BS in Equation (6).

## Appendix C. Proof of Lemma 3

Due to $W_{l}$ and $N_{l}$ can be seen as constant and $f_{l}\left(r\right)$ can be obtained based on Equation (6), we can deduce the closed-form expression of the average achievable downlink rate of UE *l* as:

(A8) $$\begin{array}{c} R_{l}=\frac{W_{l}}{N_{l}}E\left[\log_{2}\left(1+\gamma_{l}\right)\right]\\ \;\;\;\;\;=\frac{W_{l}}{N_{l}}\int_{0}^{\infty }E\left[\log_{2}\left(1+\frac{\rho_{l}P_{l}hr_{l}^{-\alpha }}{{\rho^{\prime }}_{l}I_{m}+I_{p}+\sigma^{2}}\right)\right]f_{l}\left(r\right)dr, \end{array}$$

where $P_{l}$ is full transmission power of serving BS of user type *l*, $r_{l}$ represents the distance between UE *l* and its serving BS. $\rho_{l}$ is determined as $\rho_{l}=\rho$ when l∈{pm} and ${\rho_{l}}^{\prime }$ is determined as ${\rho_{l}}^{\prime }=\rho$ when l∈{pm,pp}. Otherwise $\rho_{l}={\rho_{l}}^{\prime }=1$. Considering the complexity of Equation (A8), we can deduce $E\left[\log_{2}\left(1+\gamma_{l}\right)\right]$ first as Equation (A9).

(A9) $$\begin{array}{c} E\left[\log_{2}\left(1+\frac{\rho_{l}P_{l}hr_{l}^{-\alpha }}{{\rho^{\prime }}_{l}I_{m}+I_{p}+\sigma^{2}}\right)\right]\overset{\left(a\right)}{=}\int_{0}^{\infty }\mathrm{Prob}\left[\log_{2}\left(1+\frac{\rho_{l}P_{l}hr_{l}^{-\alpha }}{{\rho^{\prime }}_{l}I_{m}+I_{p}+\sigma^{2}}\right)>t\right]dt\\ \;\;\;\;\;\;\;\;\;\;\;\;\;\;\;\;\;\;\;\;\;\;\;\;\;\;\;\;\;\;\;\;\;\;\;\;\;\;\;=\int_{0}^{\infty }\mathrm{Prob}\left[h>\left(2^{t}-1\right)\left({\rho^{\prime }}_{l}I_{m}+I_{p}+\sigma^{2}\right)r_{l}^{\alpha }\rho_{l}^{-1}P_{l}^{-1}\right]dt\\ \;\;\;\;\;\;\;\;\;\;\;\;\;\;\;\;\;\;\;\;\;\;\;\;\;\;\;\;\;\;\;\;\;\;\;\;\;\;\;\overset{\left(b\right)}{=}\int_{0}^{\infty }\exp\left[-\left(2^{t}-1\right){\rho^{\prime }}_{l}I_{m}r_{l}^{\alpha }\rho_{l}^{-1}P_{l}^{-1}\right]\exp\left[-\left(2^{t}-1\right)I_{p}r_{l}^{\alpha }\rho_{l}^{-1}P_{l}^{-1}\right]\\ \;\;\;\;\;\;\;\;\;\;\;\;\;\;\;\;\;\;\;\;\;\;\;\;\;\;\;\;\;\;\;\;\;\;\;\;\;\;\;\;\cdot \exp\left[-\left(2^{t}-1\right)\sigma^{2}r_{l}^{\alpha }\rho_{l}^{-1}P_{l}^{-1}\right]dt\;\\ \;\;\;\;\;\;\;\;\;\;\;\;\;\;\;\;\;\;\;\;\;\;\;\;\;\;\;\;\;\;\;\;\;\;\;\;\;\;\;\overset{\left(c\right)}{=}\int_{0}^{\infty }L_{I_{m}}\left(\tau r_{l}^{\alpha }{\rho^{\prime }}_{l}\rho_{l}^{-1}P_{l}^{-1}\right)L_{I_{p}}\left(\tau r_{l}^{\alpha }\rho_{l}^{-1}P_{l}^{-1}\right)\\ \;\;\;\;\;\;\;\;\;\;\;\;\;\;\;\;\;\;\;\;\;\;\;\;\;\;\;\;\;\;\;\;\;\;\;\;\;\;\;\cdot \exp\left[-\tau \sigma^{2}r_{l}^{\alpha }\rho_{l}^{-1}P_{l}^{-1}\right]dt,\;\;\;\;\;\;\;\;\;\;\; \end{array}$$

where (a) is derived according to $E\left(X\right)=\int_{0}^{\infty }P\left[X>x\right]dx$, (b) is obtained referring to $h∼\exp\left(1\right)$, (c) is obtained through the Laplace transform of $I_{m}$ and $I_{p}$ by setting $\tau =2^{t}-1$. Referring to [6], the Laplace transform formulations can be expressed as:

(A10) LImτrlαρl′ρl−1Pl−1=exp−πλmρl′PmρlPl22ααr2τ22αα∫Bm/ρl′ρl−1Blτ22αα∞11+xαα22dx

(A11) LIpτrlαρl−1Pl−1=exp−πλpPpρlPl22ααr2τ22αα∫Bp,lBp,lBlτBlτ22αα∞11+xαα22dx,

where $B_{l}$ denotes the association bias of the typical UE *l* to select its serving BS. When l=pp, $B_{l}=B_{p}$; otherwise $B_{l}=1$. In order to simplify the analysis, we can define variable $\beta =B_{k,l}/B_{l},k\in \left\{m,p\right\}$, where $B_{m,l}=B_{m}/\left({\rho_{l}}^{\prime }\rho_{l}^{-1}\right)$ and $B_{p,l}=\left\{\begin{array}{c} 1,\mathrm{if}\;l=up\\ B_{p},\;\mathrm{otherwise} \end{array}\right.$.

Substituting Equations (A10) and (A11) into Equation (A9), and further substituting Equation (A9) into Equation (A8), we can obtain the closed-form expression of the average achievable downlink rate of a typical UE *l* as Equation (9).
